# Ecological Isolation Maintains the Species Boundaries Between Two Sympatric *Cycas* From Southwest China

**DOI:** 10.1002/ece3.71769

**Published:** 2025-07-08

**Authors:** Fanggui Zheng, Yiqing Wang, Huihui Xi, Siyue Xiao, Xiuyan Feng, Xun Gong, Jian Liu

**Affiliations:** ^1^ Southwest Forestry University Kunming China; ^2^ Key Laboratory for Economic Plants and Biotechnology, Yunnan Key Laboratory for Wild Plant Resources Kunming Institute of Botany, Chinese Academy of Sciences Kunming China; ^3^ University of Chinese Academy of Sciences Beijing China

**Keywords:** conservation, *Cycas*, reproductive isolation, species boundaries, sympatric distribution

## Abstract

Over long‐term evolutionary processes, sympatric affinities may develop reproductive isolation mechanisms, such as temporal isolation and ecological divergence, to maintain species independence. However, due to lacking strict geographic isolation barriers, sympatrically distributed closely related species may experience interspecific gene flow and genetic introgression, which can blur species boundaries. Here we focus on two sympatrically distributed *Cycas* species along the Lancang (Mekong) River in three populations from Southwest China, *Cycas pectinata* and *C. simplicipinna* , to investigate the extent of genetic introgression between them and how they maintain species boundaries. Using 16 microsatellite markers, we first genotyped and assessed the introgression patterns between the two species and their seedlings in each population. We further compared their geographical and ecological divergence, including the fine‐scale spatial distribution, habitats, reproductive phenology, and pollinators, based on a systematic field survey across its entire range in China. We found that sympatric populations of *C. pectinata* and *C. simplicipinna* , along with their seedlings, showed no genetic admixture. Further evidence supports that the two species exhibited significant variations in habitat indicators such as slope position and soil pH. Additionally, significant differences were observed in pollinator communities and coning behavior. These findings indicate that there is no hybridization between *C. pectinata* and *C. simplicipinna* under natural conditions. Instead, they maintain species boundaries primarily through reproductive isolation driven by divergent coning times and pollinator specificity, coupled with niche differentiation. This study not only provides a representative case for understanding mechanisms of plant species boundary maintenance but also offers critical theoretical support for the reintroduction and conservation of cycads.

## Graphical abstract

The study reveals that sympatric *Cycas* species ( *C. pectinata* and *C. simplicipinna* ) maintain strict species boundaries without hybridization, driven by reproductive isolation mechanisms (divergent coning times and pollinator specificity) and ecological niche differentiation (habitat preferences, soil pH differences). These findings highlight how plants sustain species independence in the absence of geographic barriers, offering critical insights for cycad conservation and understanding evolutionary isolation mechanisms.

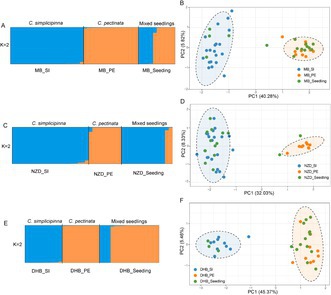

## 1 Introduction

Robust species boundary provides the critical foundation for biodiversity assessment, conservation prioritization, and targeted protection of threatened taxa (Queiroz 2007; Wiens 2007; Gong and Mamtimin 2012). Nevertheless, sympatric coexistence poses significant challenges for species boundary maintenance (Mayr 1963), as overlapping geographic ranges and shared ecological niches often drive phenotypic convergence among co‐occurring lineages (Fu et al. 2022; Qiu et al. 2023). This morphological homogenization, compounded by incomplete reproductive isolation and permeable ecological barriers, facilitates recurrent interspecific hybridization and genetic introgression between sympatric congeners (Coyne and Orr 2004). While such hybridization may ultimately drive speciation events and enhance biodiversity through reticulate evolution (Soltis and Soltis 2009), contemporary conservation biology may face immediate challenges, especially in the short‐term taxonomic consequences, which can blur species boundaries and genetic structure, complicate both systematic classifications and the development of effective conservation protocols (Mallet 2005; Soltis and Soltis 2009). Given the widespread coexistence of closely related lineages, the mechanisms maintaining species boundaries between sympatric species have become a major focus for evolutionary biologists and ecologists (Weber and Sharon 2016).

Plants are particularly instructive for studying sympatric dynamics, due to their sessile nature and constrained dispersal capacities (Levin 2000; Gastauer et al. 2015; Gao et al. 2025). In sympatric congeneric plant species, prezygotic isolation mechanisms represent important evolutionary safeguards for maintaining phenotypic distinctiveness and genomic integrity. These reproductive barriers operate through interconnected internal (behavioral and physiological constraints) and external (ecological specialization) factors, either independently or synergistically (Harrison and Larson 2014). For example, in a *Lilium* hybrid zone of northwestern Yunnan, environmental filtering eliminates maladapted hybrids through differential phenology and elevational niche divergence, effectively counteracting gene flow between three co‐occurring species (Gao et al. 2020). In *Primula*, reproductive isolation maintained through pollinator‐driven assortative mating, post‐zygotic seed viability selection, and flowering phenological differences collectively sustain species boundaries between *P. veris* and *P. vulgaris* (Keller et al. 2021). Similarly, two sympatric orchid species, *Platanthera blephariglottis* and *P. cristata* , reduce hybridization through coordinated mechanisms involving pollinator specificity, temporal flowering shifts, and differential mycorrhizal symbiont associations (Evans et al. 2023). Collectively, these cases highlight the paramount importance of multidimensional ecological isolation—particularly niche differentiation, temporal reproductive asynchrony, and mutualist‐mediated specialization—in sustaining species boundaries under sympatric conditions. Notably, current understanding derives predominantly from herbaceous angiosperm models, rendering a knowledge gap regarding boundary maintenance mechanisms in long‐lived, woody gymnosperms where different evolutionary constraints may prevail.

As one of the oldest extant gymnosperm lineages, cycads encompass two families (Cycadaceae and Zamiaceae), 10 genera and approximately 380 species, offering unparalleled insights into seed plant origins and Mesozoic adaptive radiations (Nagalingum et al. 2011; Calonje et al. 2013). The conserved morphological traits of cycads, including pinnate leaves and specialized reproductive structures, serve as critical proxies for reconstructing early land plant diversification (Chang et al. 2019, 2020; Coiro and Seyfullah 2024). Despite their phylogenetic importance, cycad conservation biology faces multiple challenges: (1) reproductive constraints of dioecy, and extremely slow reproductive turnover coupled with obligate dependence on specialist insect mutualists create critical demographic bottlenecks (Jones 2002; Terry et al. 2005, 2007; Toon et al. 2020; Salzman et al. 2023); (2) anthropogenic pressures, such as accelerated habitat fragmentation and rampant illegal trading, collectively exacerbate extinction risks, with current threat levels surpassing those of all other seed plant groups (Xi et al. 2022; Calonje et al. 2013–2025). These challenges underscore the urgent need to clarify the reproductive isolation mechanisms amidst modern anthropogenic disturbances and climate change. In this context, studies on reproductive phenology of cycads have received increasing attention: findings suggest that reproductive events in cycads are influenced by precipitation seasonality (Segalla et al. 2022), and sympatric cycads may experience synchronous reproductive timing for potential hybridization (Martínez‐Domínguez et al. 2022). However, these observations are majorly focused on the shifts of reproductive phenology, highlighting a significant knowledge gap regarding reproductive isolation mechanisms within cycad lineages.

The genus *Cycas* (Cycadaceae), comprising over 120 species, is majorly distributed across tropical and subtropical regions (Liu et al. 2024). China represents a key biodiversity hotspot for this genus, harboring more than 20 species with about 15 endemic to Yunnan Province—the country's richest cycad refuge (Xi et al. 2022). The *Cycas* in Yunnan is typically characterized by its riverine distribution habit, with numerous endemic species confined to specific watersheds. For instance, *C. diannanensis* and *C. hongheensis* are restricted to the Red River Basin in China (Liu et al. 2015, 2016), while *C. tanqingii* persists solely in the Xiaoheijiang River Basin of Lvchun County (Xi et al. 2022), and *C. guizhouensis* occupies the Nanpan River Basin (Zheng et al. 2017). While these hydrological boundaries typically enforce strong geographic isolation, minimizing interspecific contact and sympatry, complete reproductive isolation in *Cycas* remains elusive. Artificial hybridization experiments between *C. sexseminifera* and *C. panzhihuaensis* have produced fertile hybrids (Pan 2014), and natural hybridization or introgression may occur between parapatric species in wild populations (Tao et al. 2021; Xi et al. 2022; our unpublished data), as facilitated by shared pollinators such as some Coleoptera beetles and Curculionidae weevils (Tang et al. 2020; Hsiao et al. 2023).

**FIGURE 1:**
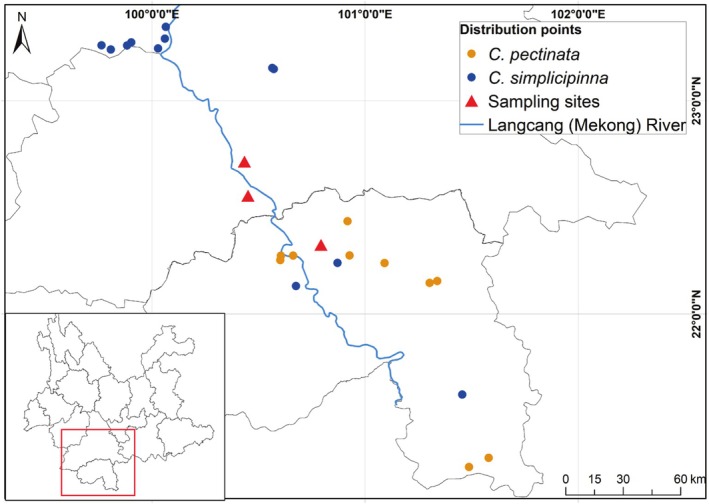
Distribution of *C. simplicipinna* and *C. pectinata* from the Lancang (Mekong) River basin of Southwest China.

**FIGURE 2:**
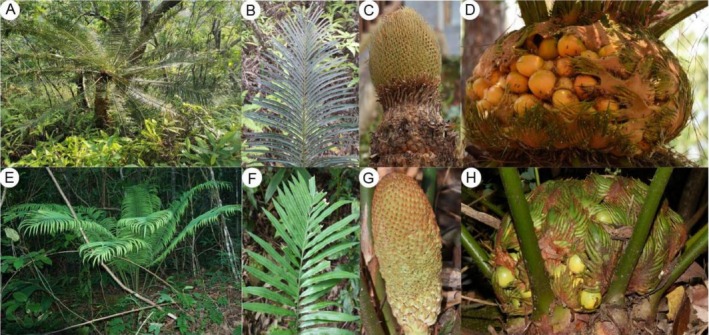
Morphology and habitats of *C. pectinata* and *C. simplicipinna* . (A–D) Represent the adult plant of *C. pectinata* and its habitat, leaf, male and female cone respectively; (E–H) represent the adult plant of *C. simplicipinna* and its habitat, leaf, male and female cone, respectively.

In this study, we investigated hybridization dynamics and species boundary maintenance between *Cycas pectinata* and *C. simplicipinna* across three sympatric populations in Xishuangbanna and Pu'er, southwestern China. Both species exhibit distributions along the Lancang (Mekong) River basin in China (Figure 1), mirroring the riverine distribution patterns characteristic of many *Cycas* taxa. Their sympatric occurrence within this shared watershed facilitates occasional cohabitation of identical microhabitats. Phylogenetic analyses confirm that *C. pectinata* and *C. simplicipinna* occupy distinct sections within *Cycas* (i.e., section *Indosinenses* and *Stangerioides*), demonstrating clear species delimitation and evolutionary distinctiveness (Liu et al. 2024). However, interspecific hybridization between *Cycas* sections was shown to be feasible (Pan 2014). Through field survey, we found that while adult plants demonstrate pronounced morphological divergence in stem, leaflets, microsporophyll, and megasporophyll (Figure 2), seedlings in mixed populations show indistinguishable vegetative morphology, obscuring hybrid identification and complicating conservation prioritization (Draper et al. 2021).

To resolve these ambiguities, we integrated population genetic approaches to quantify genetic admixture across adult and seedling cohorts. By assessing the habitat niche variation, pollinator assemblage variation, and coning phenological differences of the two species, we aim to elucidate mechanisms underpinning their reproductive isolation. Through these multidisciplinary data, our purpose is to examine: (1) whether natural hybridization occurs in co‐occurring *C. pectinata* and *C. simplicipinna* ; and (2) which reproductive and ecological mechanisms primarily sustain their taxonomic integrity despite sympatric coexistence.

## 2 Materials and Methods

### 2.1 Field Surveys and Sample Collection

Field surveys were conducted on populations of *C. simplicipinna* and *C. pectinata* in Xishuangbanna and Pu'er, Yunnan, China (Figure 1). As *Cycas* pollinators are associated with male cones for most of their life cycles (Tang et al. 2020), we collected the phenological information of male plants during field surveys by recording the pollen releasing dates of microstrobilus (male cone). Based on a prior survey (Wang et al. 2021), we also collected habitat parameters for both *Cycas* species, including altitude, slope aspect, slope gradient, position on slope, canopy density, vegetation coverage, and soil physicochemical properties (e.g., C, N, P, K content, and pH). Differences of these habitat variables between the two species were compared using *t*‐tests and visualized using the *ggplot2* package (Wickham 2016) in R v4.1.0 (R Core Team 2021).

For the three populations with mixed species (Table 1), line transect surveys were implemented. The line transect was 20 m wide and about 1000 m long. We recorded the GPS coordinates for all *Cycas* individuals along the line transect regions. All seedlings that could not be identified based on morphological characteristics were collected for subsequent identification and analysis. For adult plants, we randomly selected a total of 9–20 individuals along the line transects from each population (Table 1), with a total of 125 samples collected for genetic analysis.

**TABLE 1:** Specific information and the number of samples at the sampling sites of the three populations.

Population code	Locality	N/(°)	E/(°)	Altitude/m	Sample/*N*
MB	Mangba Village, Simao Port Town, Pu'er City	22.71	100.43	1340	*C. pectinata* : 10 *C. simplicipinna* : 20 Mixed seeding: 15
NZD	Nuo Zha Du Town, Lancang Lahu Autonomous County, Pu'er City	22.55	100.45	1251	*C. pectinata* : 9 *C. simplicipinna* : 21 Mixed seeding: 14
DHB	Dahuangba Village, Jinghong City, Xishuangbanna Prefecture	22.32	100.79	1056	*C. pectinata* : 10 *C. simplicipinna* : 10 Mixed seeding: 16
Total					125

### 2.2 SSR Genotyping

Genomic DNA was extracted from silica‐dried samples using a modified CTAB method (Doyle 1991). DNA concentration was quantified using a Qubit fluorometer, and samples were stored at −20°C. A total of 16 simple sequence repeat (SSR) primer pairs (see Table S1) were selected from published studies for PCR amplification. Amplified products were genotyped using an ABI 3730XL DNA Analyzer. Fragment sizes were analyzed with GeneMapper v5.0 (Applied Biosystems), and data were processed using GenAlEx v6.5 (Peakall and Smouse 2012).

### 2.3 Genetic Structure Analysis

The processed SSR data were analyzed using Structure v2.3.4 (Pritchard and Stephens 2000) to assess genetic clustering in three mixed populations. Parameters included a burn‐in period of 10,000, MCMC replicates of 100,000, and *K*‐values ranging from 1 to 10 (10 iterations per *K*). The optimal *K*‐value was determined using Structure Harvester (Earl and Vonholdt 2012). CLUMPP v1.1.2 (Jakobsson and Rosenberg 2007) and distruct v1.1 (Rosenberg 2004) were used for cluster visualization. We also performed principal component analysis (PCA) analyses based on SSR data using the *poppr* package v2.9.6 (Kamvar et al. 2015) in R v4.1.0, with results visualized by the *ggplot2* package. Furthermore, we used GenAIEx v6.5 to conduct the Analysis of molecular variance (AMOVA) to estimate the genetic variation and heterogeneity among the investigated populations and between species of *C. pectinata* and *C. simplicipinna* .

### 2.4 Spatial Distribution Analysis

To visualize the fine‐scale distribution pattern of the two sympatric species, the relative positions of *C. pectinata* and *C. simplicipinna* in three different populations were represented using ArcGIS v10.6 software, based on the GPS data obtained from the survey and the species delimitation results from the Structure analysis and PCA. Besides, based on the coordinates of each individual, we calculated the convex hull area size of each species in different populations in R v4.1.0 (R Core Team 2021); then we calculated the Morishita's index (Morisita 1967) and the nearest neighbor index (NNI, Clark and Evans 1954) of the two species among three sampling line transects to assess their degree of spatial dispersion. The Morisita's index was calculated using Formula (1):

(1) $$I_{\delta }=\frac{Q\sum_{i=1}^{Q}n_{i}\left(n_{i}-1\right)}{N\left(N-1\right)}$$

where *Q* is the total grid cells in the study area (the grid division was 0.0005° × 0.0005°, which is approximately 55 m × 55 m to ensure about 1–5 individuals within a grid); *n*_*i*_, number of individuals counted in the *i*‐th grid cell; and *N*, sum of all individuals across all quadrats.

The NNI was calculated using Formula (2):

(2) $$\mathrm{NNI}=\frac{{\bar{D}}_{\mathrm{obs}}}{{\bar{D}}_{\exp}}$$

where ${\bar{D}}_{\mathrm{obs}}$ is the observed mean nearest neighbor distance and ${\bar{D}}_{\exp}$ is the expected mean nearest neighbor distance under complete spatial randomness.

### 2.5 Pollinator Identification and Reproductive Phenology Analysis

Cycads typically exhibit brood‐site pollination, with pollinators primarily active within male cones (Tang et al. 2020). Therefore, during field surveys in the coning months, we investigated the insects from at least one male plant for each species in all three investigated populations. Pollinators observed in the microstrobilus of different populations were collected for further identification and comparison. Random visitors in the microstrobilus were excluded, and the potential pollinators shared in different male cones from the same species were collected and then preserved in 100% ethanol. Pollinator specimens were identified to genus level through a KEYENCE VHX‐5000 microscope, based on morphological comparisons with collections and taxonomic keys described in previous studies (Skelley et al. 2017; Tang et al. 2020). Species‐level identification was not attempted in this study, due to: (1) incomplete taxonomic descriptions of regional insect fauna, and (2) the potential presence of undescribed species within these genera. Voucher specimens are deposited in Kunming Institute of Botany, Chinese Academy of Sciences.

We compiled the reproductive phenology data of *C. pectinata* and *C. simplicipinna* from our field surveys, digitized herbarium records (Chinese Virtual Herbarium, www.cvh.ac.cn; Chinese Field Herbarium, https://www.cfh.ac.cn), and reports from reserve rangers (details described in Supporting Information). We only focused on the mature male plants from these sources and collected the pollen‐releasing dates to represent the coning records. Based on these data, we generated kernel density plots using *ggplot2* (Wickham 2016) to generate the frequency of coning for the two species.

## 3 Results

### 3.1 Genetic Structure Analyses

**FIGURE 3:**
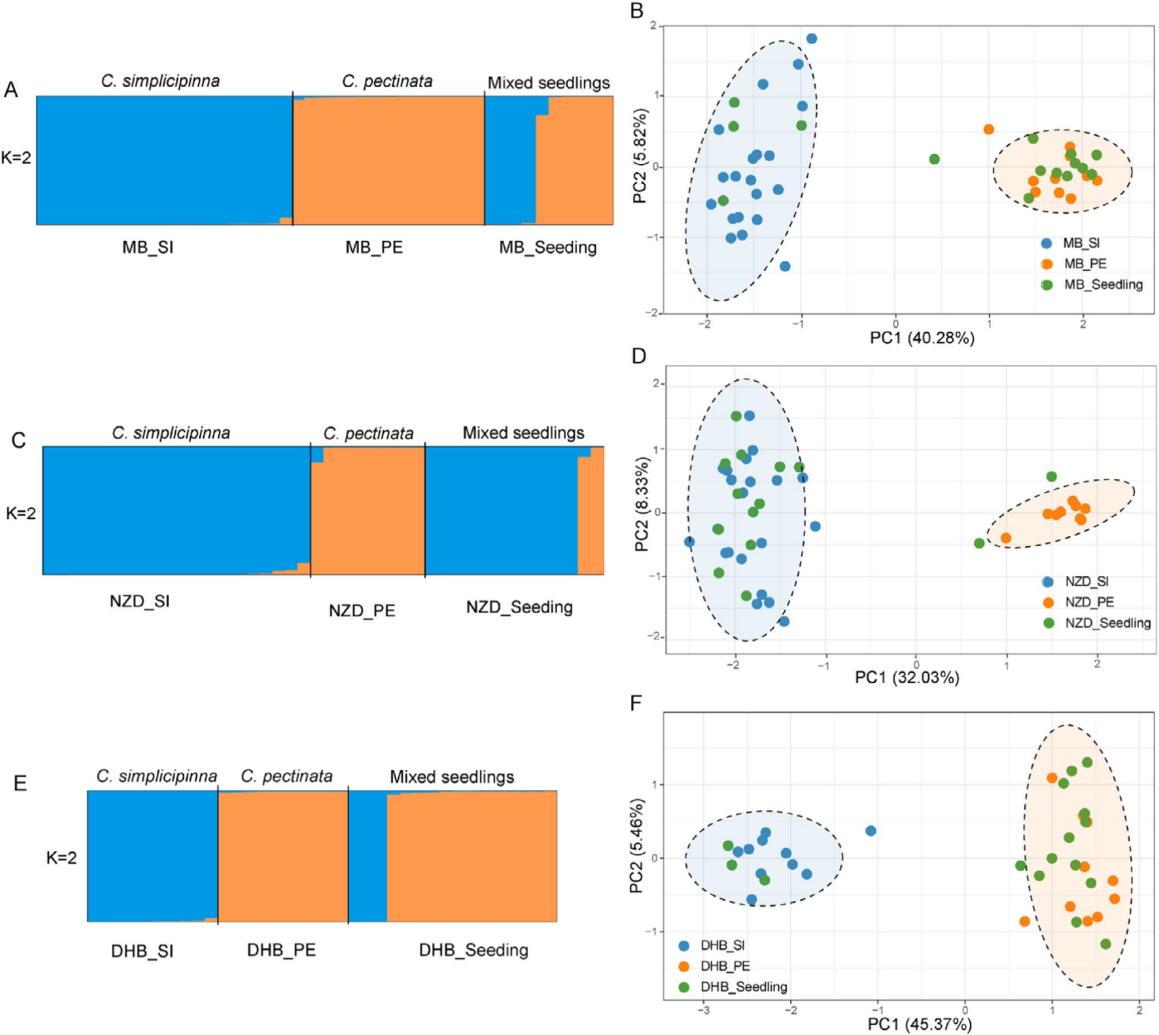
Genetic structure of *C. simplicipinna* and *C. pectinata* as well as their seedlings in three sympatric populations inferred from Structure and PCA analyses. (A‐B) Mangba population (MB); (C‐D) Nuozhadu population (NZD); (E‐F) Dahuangba population (DHB); PE, *C. pectinata*; SI, *C. simplicipinna* . In the PCA diagram, the dashed circles represent the interval at the 95% confidence level.

Based on SSR data, the Structure analyses all revealed the optimal grouping occurred at *K* = 2 (Figure S1). Under this grouping, the three mixed populations of *C. simplicipinna* and *C. pectinata* exhibited significant genetic differentiation (Figure 3). Both species displayed highly independent genetic compositions within their respective populations, with no significant introgression detected. The seedling populations were also clearly separated into two distinct genetic clusters, indicating that these seedlings strictly belonged to either *C. simplicipinna* or *C. pectinata* , with almost no individuals showing mixed genetic compositions or intermediate types. Additionally, Structure analysis across all samples identified an optimal *K* value of two. At this *K* value, individuals clustered into two genetic groups corresponding to the two species, with limited admixture observed in a few samples (Figure S2)—consistent with prior population‐level analyses.

The PCA results for adult plants and mixed seedlings of the two *Cycas* species showed that, across the three populations of NZD, MB, and DHB, the first principal component explained 32.03%, 40.28%, and 45.37% of interspecific genetic variation, respectively, while the second principal component accounted for 8.33%, 5.82%, and 5.46% variation, respectively. In all populations, *C. pectinata* and *C. simplicipinna* were distinctly divided into two independent genetic clusters, with most mixed seedlings assigned to these two groups at a 95% confidence level (Figure 3B,D,F).

The AMOVA analysis revealed that most variations are within populations, with inter‐population variation accounted for 7% and 13% of the total variation for *C. simplicipinna* and *C. pectinata* , respectively (Table S2). The *F*‐statistics (*F*_st_) indicate significant degrees of genetic differentiation among the populations of the two species, while the differentiation is greater in *C. pectinata* than *C. simplicipinna* (Table S2), suggesting more significant gene flow among *C. simplicipinna* populations. Besides, a higher heterozygosity (*H*_o_ = 0.426, *H*_e_ = 0.518) was found in *C. simplicipinna* than in *C. pectinata* (*H*_o_ = 0.352, *H*_e_ = 0.501, Table S3).

### 3.2 Spatial Distribution Patterns in Sympatric Populations

**FIGURE 4:**
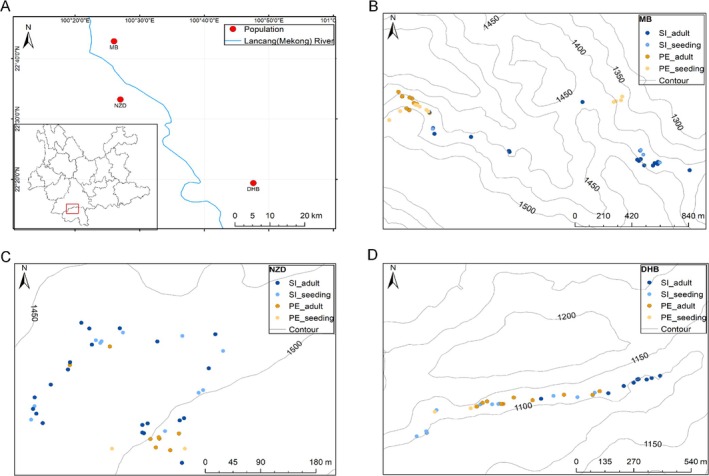
Geographical location of the three sympatric populations and the distribution of plants within the populations (A). MB (B), NZD (C), and DHB (D) represent the three populations where *Cycas* species coexist (Table 1). PE, *C. pectinata* ; SI, *C. simplicipinna* . The identified seedlings were marked in a similar color to their adult species.

The three focal populations are located on both banks of the Lancang River in southwestern Yunnan Province, within a predominantly tropical climate zone (Figure 4A). Molecular identification of seedlings revealed species‐specific spatial distribution patterns within each population. In the MB population, *C. pectinata* was primarily distributed on the northwestern side of the line transect, while *C. simplicipinna* occupied the south, with little overlap between the two species. Seedlings in this population clustered near their maternal plants, showing almost no spatial overlap with the other species (Figure 4B). In contrast, the NZD population exhibited distinct segregation: *C. pectinata* dominated the south, whereas *C. simplicipinna* was concentrated in the northern area, with a notable overlap zone in the south that may represent a transitional boundary. Similarly, seedlings here were strictly associated with their respective maternal plants, lacking apparent overlap (Figure 4C). In the DHB population, *C. pectinata* was distributed centrally along the transect, while *C. simplicipinna* occupied both eastern and western margins, showing partial overlap. Notably, *C. simplicipinna* seedlings in this population were more dispersed, with some individuals found proximal to adult *C. pectinata* (Figure 4D).

Spatial analyses based on line transects revealed that *C. simplicipinna* occupied larger distribution areas than *C. pectinata* across all populations (Table S4). Both species exhibited aggregated distribution patterns (Morisita's index > 1; NNI < 1) in all populations (Table S4). Notably, *C. pectinata* showed significantly lower NNI values than *C. simplicipinna* in MB and DHB (but not NZD), indicating stronger spatial clustering in these two populations. While Morisita's indices were comparable between species (Table S4), *C. pectinata* displayed marginally higher aggregation in NZD and DHB.

### 3.3 Variations of Habitat Characteristics Between the Two *Cycas* Species

**FIGURE 5:**
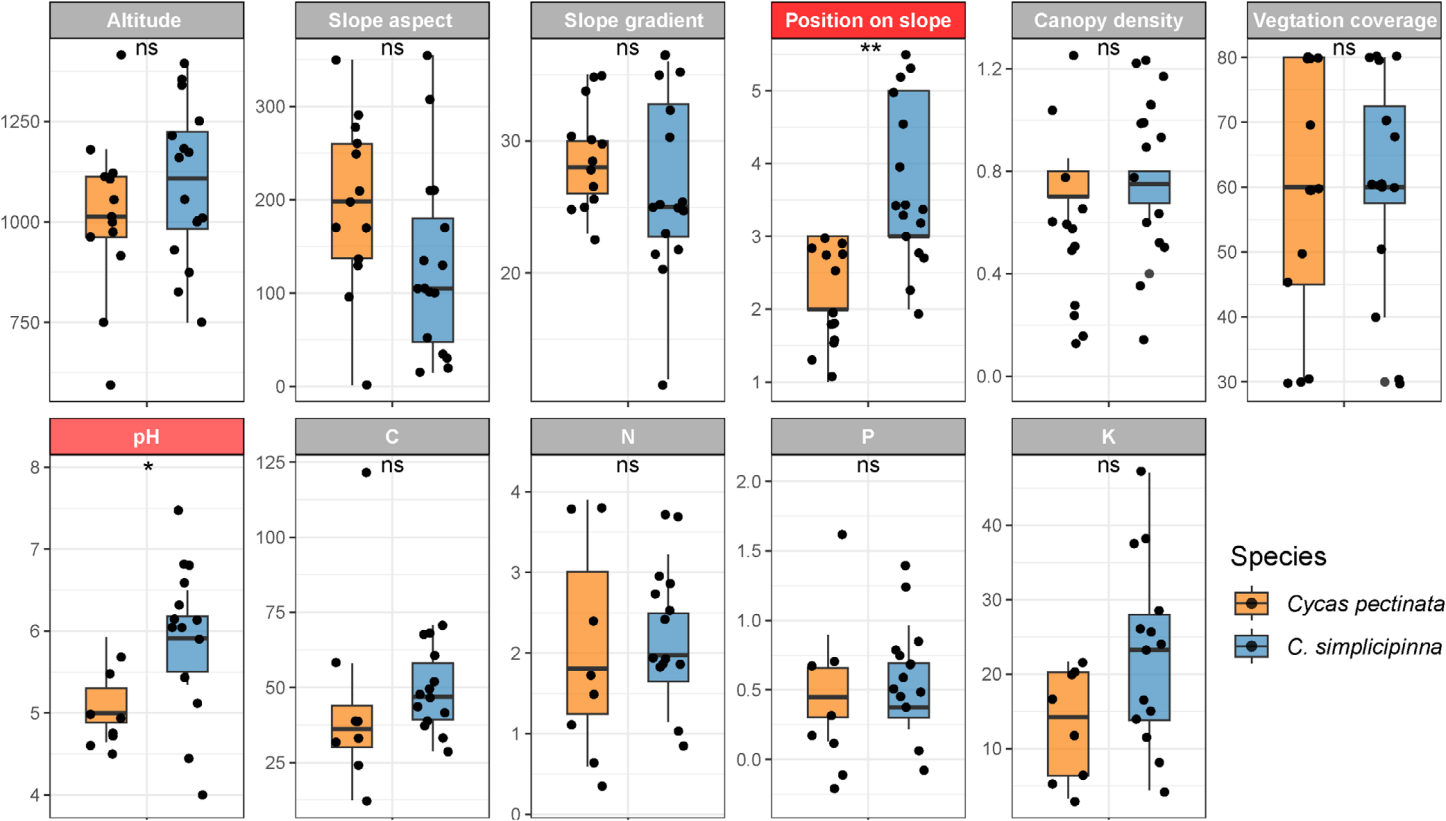
Habitat differences of the populations where *C. pectinata* and *C. simplicipinna* distributed in the Yunnan Province of Southwest China. For the colors and symbols on the top of the boxplot: gray, habitat variables with no significant difference; light red (*), with significant difference (*p* < 0.05); dark red (**), with extremely significant difference (*p* < 0.01).

Based on the comparative analysis of habitat indicators (elevation, slope aspect, slope gradient, slope position, canopy density, vegetation cover) and soil physicochemical properties (pH, C, N, P, K) between 13 *C. pectinata* and 16 *C. simplicipinna* populations (Table S5), we found the two species showed no significant differences in elevation, slope aspect, slope gradient, canopy density, or vegetation cover. However, *C. simplicipinna* occupied significantly higher slope positions compared to *C. pectinata* (Figure 5). Soil analysis indicated no notable differences in the composition of major nutrients (C, N, P, K), but a significant divergence in soil pH was observed, with *C. pectinata* favoring more acidic soils (Figure 5).

### 3.4 Pollinating Insects and Distribution of Coning Time

**FIGURE 6:**
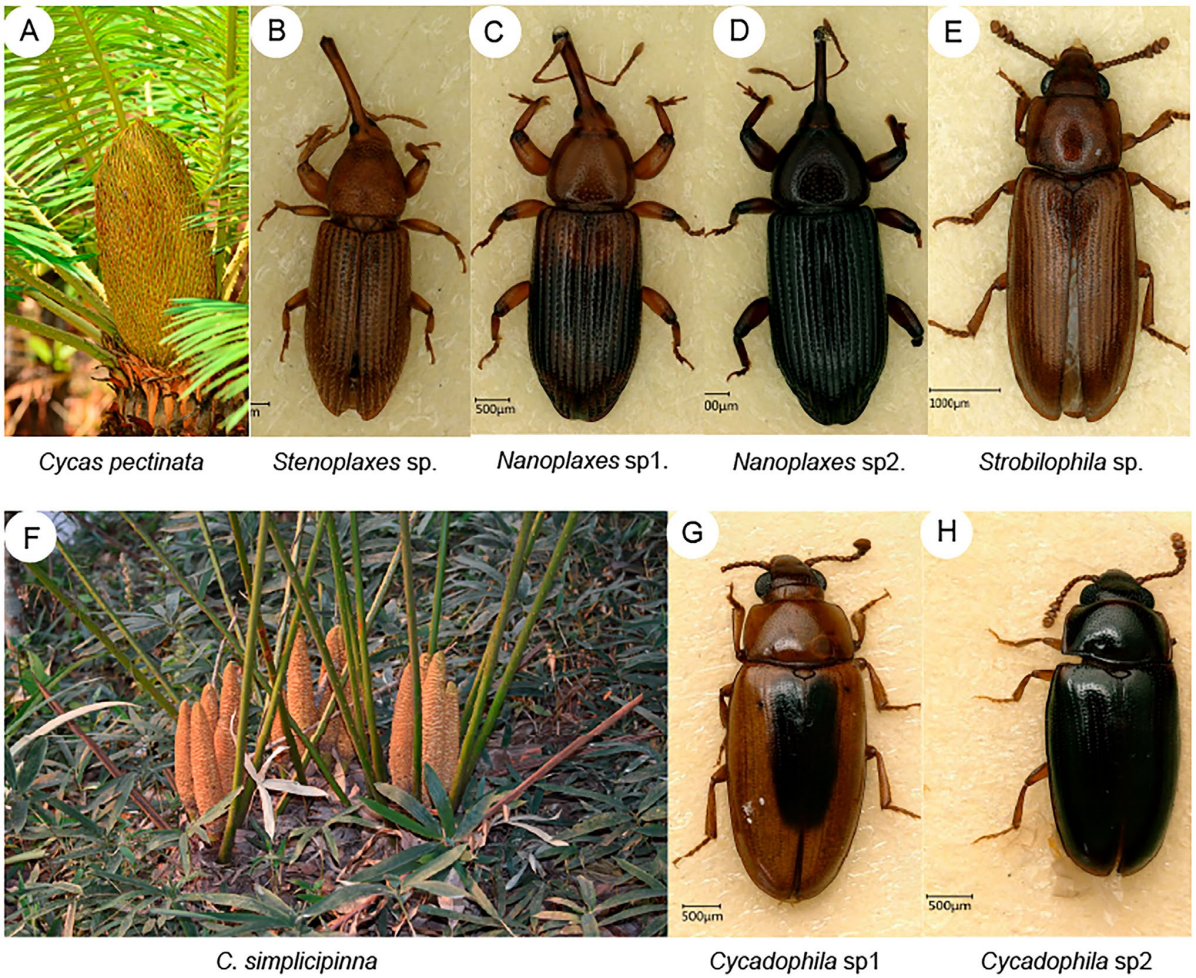
Comparison of active insects (pollinators) from the male cones of the two *Cycas* species. (A–E) Male cone and insects from *Cycas pectinata* cones; (F–H) male cones and beetles from *C. simplicipinna* cones.

We obtained potential pollinators from at least one male cone for each species in all three investigated populations. Morphological identification of these potential pollinating insects showed that *C. pectinata* was pollinated by four species belonging to three genera from two families: the weevil family Curculionidae (genera *Stenoplaxes* and *Nanoplaxes*) and the pleasing fungus beetle family Erotylidae (genus *Cycadophila* subgenus *Strobilophila*, Figure 6A–E). Among these, weevils (Curculionidae) dominated as the primary pollinators, while *Strobilophila* beetles occurred at lower frequencies. In contrast, *C. simplicipinna* is pollinated by only two species, both belonging to the *Cycadophila* subgenus *Cycadophila* within the Erotylidae family (Figure 6F–H). Notably, the pollinators from the two *Cycas* hosts are different and do not appear to share a common pollinator.

**FIGURE 7:**
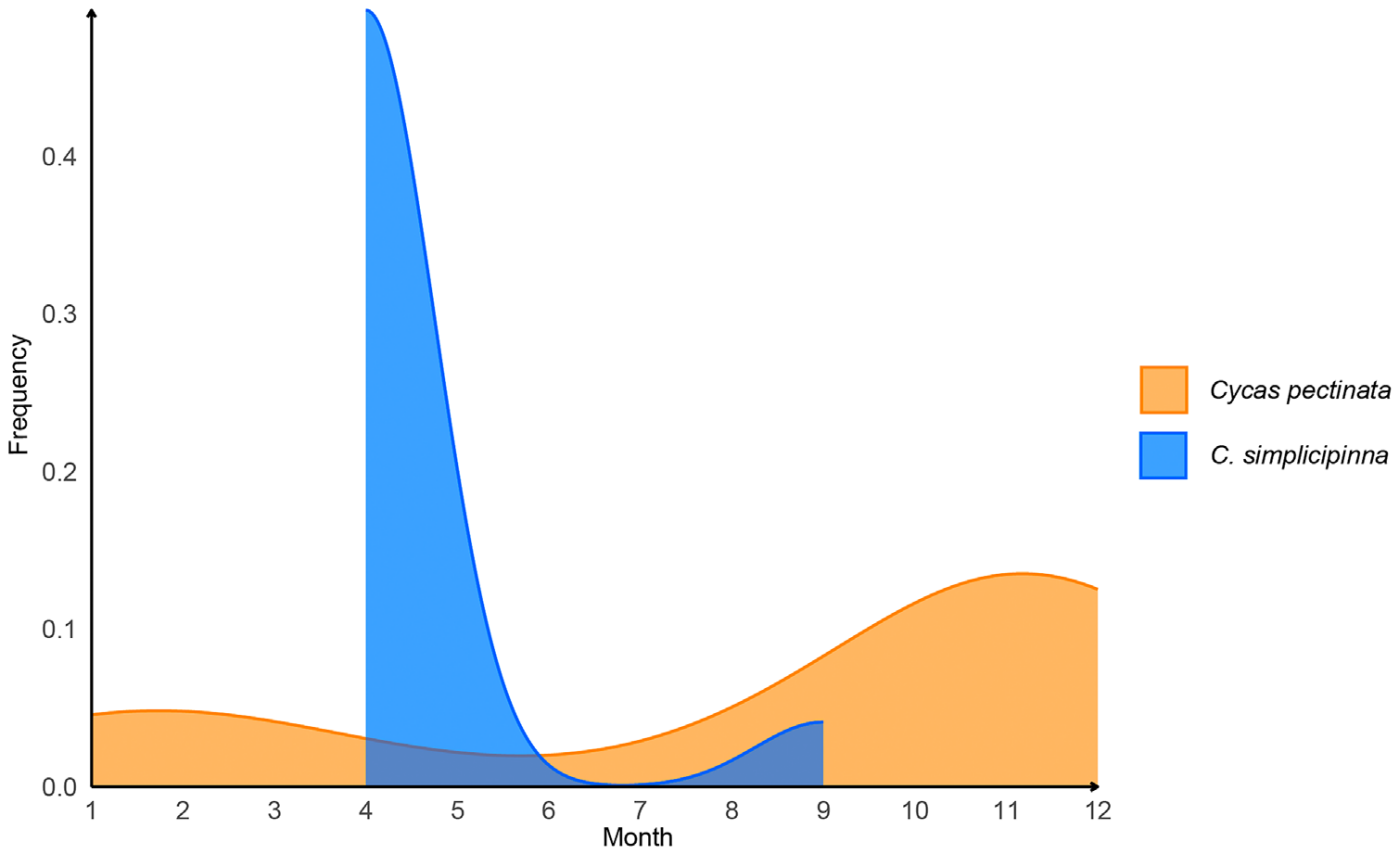
Comparison of the coning frequency between *C. pectinata* and *C. simplicipinna*.

Regarding coning behavior (Figure 7), the pollen releasing of microstrobilus in two species exhibited partially overlapping coning periods but distinct frequency peaks. Specifically, *C. pectinata* showed a dispersed coning pattern, with cones observed across multiple months. However, the male cones peak in two periods: a major peak between November and December, and a secondary peak from January to February. Notably, almost no male cone was observed for *C. pectinata* between May and July. In comparison, *C. simplicipinna* exhibited a concentrated microstrobilus coning period confined to April to September, with peaks in April to May. Minor coning events occurred sporadically in May to June and August to September. Despite this, no *C. simplicipinna* male cone was recorded during *C. pectinata* 's peak months (November to February of the next year).

## 4 Discussion

### 4.1 Mechanisms Maintaining Species Boundaries Between *C. pectinata* and *C. simplicipinna*

The genetic structure and PCA analyses of *C. pectinata* , *C. simplicipinna* , and their seedlings across three sympatric populations reveal pronounced genetic divergence between these species, with no detectable genetic admixture in seedlings from mixed populations. These findings demonstrate robust reproductive isolation, despite their close spatial proximity and absence of geographic barriers. Interspecific hybridization between different *Cycas* sections has been reported previously (Pan 2014), suggesting postzygotic isolation mechanisms (e.g., physiological incompatibility or hybrid inviability) are unlikely to account for their reproductive isolation. Instead, our results indicate the species boundaries between sympatric *Cycas* appear to be maintained through prezygotic isolation mechanisms mediated by pollinator specificity, phenological divergence, and ecological niche differentiation.

Cycads often exhibit obligate pollination mutualisms with specialized insect partners (Terry et al. 2005; Salzman et al. 2023). In *Cycas*, pollinators primarily involve beetles from the Erotylidae and Curculionidae families, which display section‐specific associations (Toon et al. 2020; Hsiao et al. 2023). Our observations reveal distinct pollinator assemblages between the focal species: *C. pectinata* interacts with a more diverse pollinator community spanning two families and three genera, predominantly Curculionid beetles, with rare visits from Erotylidae (*Cycadophila* subgenus *Strobilophila*) that likely represent opportunistic predators rather than dedicated pollinators. In contrast, *C. simplicipinna* exclusively relies on a single Erotylidae subgenus (*Cycadophila*, Figure 6G,H). This stark contrast in pollinator associations at both family and subgenus levels establishes behavioral isolation, effectively preventing interspecific pollen transfer.

Furthermore, temporal isolation arises from their contrasting coning phenologies. *Cycas pectinata* exhibits a bimodal coning pattern, with primary coning in November to December and a secondary peak in January to February, but almost no coning from May to July. Conversely, *C. simplicipinna* cones exclusively from April to September, completely avoiding *C. pectinata* 's primary reproductive window, with at least 2 months' interval between species (Figure 7). While the phenological data in this study was focused on male cones, our field surveys and observations on cultivated *Cycas* show the receptivity of ovuliferous strobili (female cone) normally begins days after pollen release and lasts less than 2 weeks. Therefore, the dioecious and dichogamous nature of *Cycas*, combined with brief pollen receptivity periods, ensures that pre‐existing pollinator specificity and phenological divergence collectively eliminate cross‐pollination opportunities. This synergistic interaction between pollinator‐mediated behavioral isolation and temporal niche partitioning establishes species boundaries by preventing genetic introgression, exemplifying how specialized mutualisms and reproductive timing strategies maintain isolation in sympatric cycads.

### 4.2 Ecological Isolation and Species Coexistence Between *C. pectinata* and *C. simplicipinna*

Our study identified significant ecological divergence in slope position preferences and soil pH adaptation between *C. pectinata* and *C. simplicipinna* , suggesting niche divergence partially facilitates their sympatric coexistence (Wang et al. 2021). Similar patterns occur in other plant systems, including *Habenaria davidii* and *H. fordii* (Zhang and Gao 2017), *H. limprichtii* and *H. delavayi* (Zhang et al. 2022), as well as *Geodorum densiflorum* and *G. eulophioides* (Zhu et al. 2023), where measurable habitat differentiation enables co‐occurrence. While ecological isolation via niche divergence may represent a widespread mechanism for sympatric persistence across taxa, its efficacy depends on synergistic interactions with reproductive barriers. For instance, ecologically distinct *Melastoma penicillatum* and *M. dendrisetosum* hybridize with widespread congeners ( *M. candidum* , *M. sanguineum* ) in Hainan Island of China, due to incomplete reproductive isolation and anthropogenic pressures (Zhou et al. 2017), underscoring the necessity of complementary mechanisms like phenological or pollinator‐mediated isolation.

Within sympatric populations, *C. simplicipinna* generally exhibited broader spatial dispersion than *C. pectinata* (Figure 4B–D; Table S4), potentially explained by two hypotheses: (1) gravity‐mediated seed dispersal at higher slope positions (Figure 5) enhances roll‐out distances for *C. simplicipinna* , despite cycads' typically limited dispersal capacities (Wu et al. 2024); (2) rodent‐mediated transport may be impeded for *C. pectinata* due to its taller stems and larger seeds. The efficient seed dispersal of *C. simplicipinna* may result in more frequent gene flow among its populations than *C. pectinata* , as evident by lower genetic differentiation levels and higher heterogeneity in *C. simplicipinna* in the three investigated sympatric populations of this study (Tables S2 and S3). Remarkably, both species maintain extensive distributions across the Lancang‐Mekong Basin (Feng et al. 2014; Khuraijam and Singh 2020; Calonje et al. 2013–2025), contrasting with narrowly endemic *Cycas* congeners (Liu et al. 2015; Feng et al. 2016, 2017; Xiao et al. 2020; Xi et al. 2022; Wu et al. 2024). This sympatry likely reflects secondary range overlap following tens of millions of years of independent evolution (Liu et al. 2024), where pre‐established reproductive barriers—rather than geographic separation—sustain species integrity.‌.

### 4.3 Conservation Implications and Prospects

Based on the above findings, current *Cycas* conservation strategies that emphasize in situ protection, ex situ cultivation, and reintroductions (Xi et al. 2022) require refinement for sympatric taxa. First, while natural populations of *C. pectinata* and *C. simplicipinna* exhibit strong reproductive isolation, the potentially climate‐driven phenological shifts in the future necessitate a long‐term coning monitoring to detect temporal overlap risks. Second, in the management of mixed populations, molecular screening should be applied to supplant the error‐prone morphological identification of seedlings. Third, future reintroduction practices should make stringent spatial plans to prevent artificial hybridization between phylogenetically proximate or phenologically convergent species (Tao et al. 2021; Xi et al. 2022), particularly avoiding co‐location within protected zones.

To our knowledge, this study provides the first case in cycads demonstrating how sympatric species maintain reproductive boundaries through integrated mechanisms. Our findings demonstrate that ecological divergence coupled with reproductive isolation barriers (phenological mismatch and pollinator specificity) significantly limits hybridization between these geographically proximate species. This pattern reveals key diversification mechanisms in cycads and warrants investigation in other cycad lineages with sympatric (e.g., Martínez‐Domínguez et al. 2016) or parapatric distributions (Zheng et al. 2017). Nevertheless, our study has several limitations, including restricted sampling (Yunnan populations only), transect‐based spatial co‐existence analyses lacking fine‐scale resolution, and short‐term phenological data. Future studies should prioritize comprehensive range‐wide genomic surveys and decadal‐scale phenological tracking to elucidate *Cycas* coexistence dynamics, thereby strengthening the scientific basis for conserving these evolutionarily distinct but ecologically overlapping cycads.

## 5 Conclusion

This study demonstrates that *C. pectinata* and *C. simplicipinna* maintain strict species boundaries in sympatry through synergistic prezygotic isolation mechanisms. Pollinator specificity—mediated by divergent beetle assemblages at family and subgenus levels—combined with non‐overlapping coning phenologies (bimodal vs. seasonal) and ecological niche differentiation (slope position, soil pH) collectively prevent interspecific gene flow. Hence, conservation strategies must prioritize molecular screening for seedling identification, long‐term phenological monitoring under climate change, and cautious reintroduction protocols to avoid artificial hybridization risks. Despite limitations in spatial and temporal sampling, this work establishes a framework for understanding how specialized mutualisms, temporal niche divergence, and microhabitat specialization sustain species integrity in ancient gymnosperms, offering insights into sympatric coexistence mechanisms across plant lineages.

## Author Contributions

**Fanggui Zheng:** data curation (lead), formal analysis (equal), investigation (lead), software (equal), visualization (equal), writing – original draft (equal). **Yiqing Wang:** formal analysis (supporting), validation (supporting), writing – review and editing (supporting). **Huihui Xi:** investigation (equal), writing – review and editing (supporting). **Siyue Xiao:** conceptualization (equal), investigation (equal), writing – review and editing (equal). **Xiuyan Feng:** investigation (supporting), writing – review and editing (supporting). **Xun Gong:** conceptualization (equal), supervision (equal). **Jian Liu:** conceptualization (lead), data curation (equal), formal analysis (equal), funding acquisition (equal), investigation (equal), methodology (equal), resources (lead), supervision (lead), validation (equal), visualization (equal), writing – original draft (equal), writing – review and editing (lead).

## Conflicts of Interest

The authors declare no conflicts of interest.

## Supporting information


Data S1.

Data S2.

## Data Availability

The data generated in this study can be found in the Supporting Information.
