# Supplementary material for: Ecological Isolation Maintains the Species Boundaries Between Two Sympatric Cycas From Southwest China
Source: Ecol Evol. 2025 Jul 8;15(7):e71769. doi: 10.1002/ece3.71769 (PMC12237619; doi:10.1002/ece3.71769)
Supplement: Supplementary file 1 — Data S1. [file ECE3-15-e71769-s001.docx]

**Supplementary Information**

**Supplementary methods**

*Access of phenological information and standardization*

Phenological information was gathered from three sources:

1. Field surveys. The primary coning season for focal species was determined through repeated visits to wild populations. However, significant disparity exists in coning frequency between sexes, with males predominating and females being scarce within populations. Additionally, female coning typically begins several days after males within the same *Cycas* species but lasts substantially longer. Due to males’ greater detectability and more consistent coning duration, field surveys during the reproductive season focused on recording male coning plants. Only cones that had reached pollen release (a period from pre-release to post-release) were considered effective.
2. Herbarium records. We examined digitized *Cycas* specimens from CVH (Chinese Virtual Herbarium, www.cvh.ac.cn) and online image databases (CFH: Chinese Field Herbarium, https://www.cfh.ac.cn), and verified the species identity. We then recorded collection/photo dates exclusively from male cones showing evidence of pollen release (e.g., opened microsporangia on healthy microsporophylls or visible pollen on specimens). Collection dates for herbarium specimens and photograph timestamps were extracted from online database records.
3. Natural Reserve rangers. Rangers provided first-hand reports of pollen release events, serving as reliable phenological records.

**Table S1** Information of 16 SSR primers used in this study.

| Primer ID | Original ID | Primer Sequence | Annealing Temperature (°C) | Motif | PCR Product Size Range (bp) | References |
| --- | --- | --- | --- | --- | --- | --- |
| B1 | Cy-Tai EST-SSR01 | F:CGAGAAGTAATTTGCAAATGC | 55 | (TC)24(TG)5 | 98 | Ju et al. 2011 |
|  |  | R:TGTGAAGCTAAATAGTTGGG |  |  |  |  |
| B2 | G46 | F:CAAAAGCCCCTTGAAACC | 55 | TC | 165-173 | Yang et al. 2008 |
|  |  | R:AAGCTCGACTTGTAGATGGA |  |  |  |  |
| B3 | Cy-Tai EST-SSR05 | F:AACAGACCATGAGGACCAGG | 55 | (TGG)7N(ATA)5(ACA)3 | 232-235 | Ju et al. 2011 |
|  |  | R:GCTGGTATTCCTTAATGCAC |  |  |  |  |
| B4 | Cy-Tai EST-SSR08 | F:GAAAATGCTTTGATGTTCCC | 60 | (ATGT)4(TA)10(CA)9 | 170-177 | Ju et al. 2011 |
|  |  | R:TGGGCCAACTTTAAGCACAC |  |  |  |  |
| B5 | CY232 | F:TCTTGCTTACCCGTTTGCTT | 55 | (GT)24 | 230-240 | Cibrián-Jaramillo et al. 2008 |
|  |  | R:CTCCTCGACGTTCAATCACA |  |  |  |  |
| B6 | HL01 | F:TCCTTTCATGGACACTCTTACG | 48 | AG | 168-200 | Li et al. 2009 |
|  |  | R:ATTTATTAGATGTCTAGCTTA |  |  |  |  |
| B7 | HL08 | F:AAAACATTCCTTGCCCTGT | 56 | (TTC)12 | 228 | Li et al. 2009 |
|  |  | R:GGAGCCTGTTGAAGAGCTA |  |  |  |  |
| B8 | Cha04 | F:ACTCCTCACTCCCTCGC | 64 | (AC)17(AT)7 | 171 | Zhang et al. 2009) |
|  |  | R:CTAGCCTCTTGTTGCCATTAT |  |  |  |  |
| B9 | E004 | F:CTATCATCAGAGCCTCATTC | 54 | (AT)11 | 116-132/150 | Yang et al. 2008 |
|  |  | R:AAGTCATACATGGACAGCAA |  |  |  |  |
| B10 | Cpz8 | F:TGCTAAAGTGAACGACGAA | 55 | (AAG)7 | 287-302 | Zhang et al. 2010 |
|  |  | R:CATACTCCTTATCCCACGAAT |  |  |  |  |
| B11 | Cha05 | F:GTCTGCTAACATCTATAAA | 52 | CT | 246/175 | Zhang et al. 2009 |
|  |  | R:GATGAGCTAAGAGTCATAGTA |  |  |  |  |
| B12 | E001 | F:TGGGATTAATATTCCAGAAA | 52 | (CA)10 | 266–270 | Yang et al. 2008 |
|  |  | R:CGACGAGTCTGATGTAGGTAT |  |  |  |  |
| B13 | Cha-estssr01 | F:GATTCTTGCTCTGTTCGCTCAT | 60 | AT | 232-390 | Wang et al. 2008 |
|  |  | R:CAGAACCCCTGAACTGTCAAAC |  |  |  |  |
| B14 | HL02 | F:GGGGTTCATATCACATAAC | 50 | (GT)17(GA)11 | 147 | Li et al. 2009 |
|  |  | R:CTATAAAGAATCATCGTTCTC |  |  |  |  |
| B15 | Cha-estssr02 | F:ATAGGCTTCCTTTAGTGATGTC | 50 | CT)5(AG)4G(GA)5 | 241 | Wang et al. 2008 |
|  |  | R:GCCTTTAGTAGTATCGGATTA |  |  |  |  |
| B16 | Cha-estssr04 | F:GATGTTCCCAAATAATGTTACA | 54 | (AT)3GT(AT)9AG(AC)4 | 222 | Wang et al. 2008 |
|  |  | R:CAAGCTGCACATGCAATGA |  |  |  |  |

**Table S2** Variation and differentiation of the two *Cycas* species based on AMOVA (Analysis of Molecular Variance) results.

| **Species** | **Source** | **df** | **%** | **Fst** | **P (rand >= data)** |
| --- | --- | --- | --- | --- | --- |
| ***C. simplicipinna*** | Among Pops | 2 | 7% | 0.067 | 0.001 |
|  | Within Pops | 137 | 93% |  |  |
| ***C. pectinata*** | Among Pops | 2 | 13% | 0.128 | 0.001 |
|  | Within Pops | 107 | 87% |  |  |

**Table S3** Genetic diversity parameters inferred from three populations of *C. simplicipinna* and *C. pectinata* for different SSR loci. For abbreviations: number of samples (N), the number of alleles (Na), the effective number of alleles (Ne), the information index (I), the observed heterozygosity (Ho), the expected heterozygosity (He), unbiased expected heterozygosity (uHe), Fixation index (F).

| **Pop** | **Locus** | **N** | **Na** | **Ne** | **I** | **Ho** | **He** | **uHe** | **F** |
| --- | --- | --- | --- | --- | --- | --- | --- | --- | --- |
| ***C. simplicipinna*** | B1 | 70 | 3.000 | 1.686 | 0.715 | 0.400 | 0.407 | 0.410 | 0.017 |
|  | B2 | 70 | 3.000 | 1.155 | 0.293 | 0.114 | 0.134 | 0.135 | 0.149 |
|  | B3 | 70 | 4.000 | 1.075 | 0.192 | 0.071 | 0.070 | 0.070 | -0.025 |
|  | B4 | 70 | 10.000 | 3.137 | 1.438 | 0.214 | 0.681 | 0.686 | 0.685 |
|  | B5 | 70 | 4.000 | 2.060 | 0.832 | 0.529 | 0.514 | 0.518 | -0.027 |
|  | B6 | 70 | 4.000 | 1.192 | 0.371 | 0.171 | 0.161 | 0.162 | -0.065 |
|  | B7 | 70 | 8.000 | 3.415 | 1.378 | 0.586 | 0.707 | 0.712 | 0.172 |
|  | B8 | 68 | 12.000 | 5.090 | 1.893 | 0.721 | 0.804 | 0.809 | 0.103 |
|  | B9 | 70 | 6.000 | 3.061 | 1.261 | 0.643 | 0.673 | 0.678 | 0.045 |
|  | B10 | 70 | 6.000 | 2.663 | 1.180 | 0.557 | 0.624 | 0.629 | 0.108 |
|  | B11 | 70 | 2.000 | 1.415 | 0.469 | 0.186 | 0.293 | 0.295 | 0.367 |
|  | B12 | 70 | 9.000 | 3.528 | 1.481 | 0.543 | 0.717 | 0.722 | 0.242 |
|  | B13 | 69 | 22.000 | 9.095 | 2.505 | 0.841 | 0.890 | 0.897 | 0.056 |
|  | B14 | 70 | 12.000 | 4.947 | 1.955 | 0.657 | 0.798 | 0.804 | 0.176 |
|  | B15 | 69 | 4.000 | 1.287 | 0.472 | 0.101 | 0.223 | 0.225 | 0.545 |
|  | B16 | 70 | 10.000 | 2.451 | 1.316 | 0.486 | 0.592 | 0.596 | 0.179 |
| ***C. pectinata*** | B1 | 55 | 2.000 | 1.037 | 0.091 | 0.000 | 0.036 | 0.036 | 1.000 |
|  | B2 | 55 | 9.000 | 5.623 | 1.842 | 0.055 | 0.822 | 0.830 | 0.934 |
|  | B3 | 55 | 3.000 | 2.373 | 0.935 | 0.582 | 0.579 | 0.584 | -0.006 |
|  | B4 | 55 | 5.000 | 1.183 | 0.377 | 0.145 | 0.154 | 0.156 | 0.058 |
|  | B5 | 55 | 5.000 | 1.706 | 0.815 | 0.418 | 0.414 | 0.418 | -0.011 |
|  | B6 | 55 | 2.000 | 1.976 | 0.687 | 0.527 | 0.494 | 0.499 | -0.067 |
|  | B7 | 55 | 3.000 | 1.884 | 0.698 | 0.364 | 0.469 | 0.474 | 0.225 |
|  | B8 | 39 | 20.000 | 8.767 | 2.540 | 0.564 | 0.886 | 0.897 | 0.363 |
|  | B9 | 55 | 3.000 | 2.073 | 0.771 | 0.600 | 0.518 | 0.522 | -0.159 |
|  | B10 | 55 | 1.000 | 1.000 | 0.000 | 0.000 | 0.000 | 0.000 | #N/A |
|  | B11 | 52 | 10.000 | 5.753 | 1.976 | 0.115 | 0.826 | 0.834 | 0.860 |
|  | B12 | 55 | 8.000 | 3.232 | 1.517 | 0.745 | 0.691 | 0.697 | -0.079 |
|  | B13 | 55 | 9.000 | 3.638 | 1.487 | 0.855 | 0.725 | 0.732 | -0.178 |
|  | B14 | 17 | 6.000 | 2.664 | 1.237 | 0.118 | 0.625 | 0.643 | 0.812 |
|  | B15 | 55 | 5.000 | 2.474 | 1.141 | 0.400 | 0.596 | 0.601 | 0.329 |
|  | B16 | 55 | 5.000 | 1.230 | 0.455 | 0.145 | 0.187 | 0.189 | 0.223 |
| ***C. simplicipinna*** | **Mean** | **69.750** | **7.438** | **2.953** | **1.109** | **0.426** | **0.518** | **0.522** | **0.170** |
| ***C. pectinata*** | **Mean** | **51.438** | **6.000** | **2.913** | **1.036** | **0.352** | **0.501** | **0.507** | **0.287** |
| **Total** | **Mean** | **60.594** | **6.719** | **2.933** | **1.073** | **0.389** | **0.510** | **0.514** | **0.227** |

**Table S4** Estimated indices of distribution pattern for *C. simplicipinna* (SI) and *C. pectinata* (PE) in different populations.

| **Populations** | **NZD** | | **MB** | | **DHB** | |
| --- | --- | --- | --- | --- | --- | --- |
| Species | SI | PE | SI | PE | SI | PE |
| Area size | 47761 | 15865 | 495234 | 196499 | 61705 | 54421 |
| Morisita’s Index | 1.4355 | 1.4444 | 1.3225 | 1.2929 | 1.2436 | 1.2589 |
| NNI | 0.6811 | 0.7268 | 0.5255 | 0.1937 | 0.7640 | 0.4027 |

**Note: Area size**: the size of convex hull based on the distribution points of each species

**Morisita’s Index**: Values > 1: aggregated distribution; Values ≈ 1: random distribution; Values < 1: uniform distribution.

**NNI (Nearest Neighbor Index):** Values > 1: uniform/dispersed distribution; Values ≈ 1: random distribution; Values < 1: clustered distribution.

**Table S5** Habitat information of *Cycas pectinata* and *Cycas simplicipinna* investigated in Southwest China (From Wang et al., 2021).

| Species | Altitude | Slope aspect | Slope gradient | Position on slope | Canopy density | Vegetation coverage | pH | C | N | P | K |
| --- | --- | --- | --- | --- | --- | --- | --- | --- | --- | --- | --- |
| *Cycas pectinata* | 594 | 249 | 30 | 1 | 0.50 | 80 | NA | NA | NA | NA | NA |
| *Cycas pectinata* | 1180 | 350 | 25 | 1.5 | 0.80 | 30 | 4.80 | 31.98 | 1.31 | 0.37 | 5.47 |
| *Cycas pectinata* | 975 | 291 | 35 | 2 | 0.70 | 80 | 4.91 | 33.26 | 1.68 | 0.90 | 20.49 |
| *Cycas pectinata* | 1107 | 278 | 28 | 2 | 0.70 | 80 | 5.69 | 39.00 | 1.93 | 0.52 | 20.13 |
| *Cycas pectinata* | 750 | 96 | 25 | 3 | 0.80 | 30 | NA | NA | NA | NA | NA |
| *Cycas pectinata* | 1000 | 130 | 27 | 2 | 0.70 | 60 | 4.96 | 39.20 | 2.86 | 0.58 | 12.22 |
| *Cycas pectinata* | 962 | 170 | 30 | 2.5 | 0.70 | 45 | 4.65 | 57.95 | 3.45 | 0.33 | 16.30 |
| *Cycas pectinata* | 1013 | 260 | 28 | 1.5 | 0.80 | 30 | NA | NA | NA | NA | NA |
| *Cycas pectinata* | 916 | 170 | 35 | 3 | 0.75 | 80 | 5.03 | 121.62 | 3.90 | 1.71 | 21.65 |
| *Cycas pectinata* | 1416 | 2 | 30 | 3 | 0.40 | 60 | 5.93 | 12.45 | 0.59 | 0.13 | 6.68 |
| *Cycas pectinata* | 1122 | 137 | 26 | 2 | 0.70 | 70 | 5.18 | 24.53 | 1.06 | 0.21 | 3.33 |
| *Cycas pectinata* | 1056 | 210 | 23 | 3 | 0.60 | 60 | NA | NA | NA | NA | NA |
| *Cycas pectinata* | 1113 | 198 | 34 | 3 | 0.85 | 50 | NA | NA | NA | NA | NA |
| *Cycas simplicipinna* | 1173 | 210 | 30 | 2 | 0.80 | 70 | 4.19 | 51.68 | 2.27 | 0.42 | 14.79 |
| *Cycas simplicipinna* | 1010 | 20 | 25 | 3 | 0.80 | 30 | 4.30 | 28.91 | 1.14 | 0.22 | 4.48 |
| *Cycas simplicipinna* | 750 | 30 | 25 | 3 | 0.80 | 30 | NA | NA | NA | NA | NA |
| *Cycas simplicipinna* | 1000 | 130 | 25 | 2 | 0.70 | 60 | 6.20 | 70.72 | 3.76 | 0.91 | 28.58 |
| *Cycas simplicipinna* | 826 | 135 | 35 | 4 | 0.40 | 40 | 7.52 | 67.70 | 3.00 | 0.79 | 26.13 |
| *Cycas simplicipinna* | 1355 | 355 | 12 | 5 | 0.60 | 80 | NA | NA | NA | NA | NA |
| *Cycas simplicipinna* | 874 | 100 | 32 | 5 | 0.75 | 70 | 6.50 | 60.34 | 2.55 | 0.38 | 25.36 |
| *Cycas simplicipinna* | 1394 | 307 | 36 | 5 | 0.40 | 60 | 6.10 | 67.60 | 3.22 | 0.75 | 22.75 |
| *Cycas simplicipinna* | 1215 | 105 | 25 | 5 | 0.80 | 80 | 6.07 | 43.61 | 1.96 | 0.53 | 37.54 |
| *Cycas simplicipinna* | 1160 | 105 | 25 | 5 | 0.80 | 80 | 6.13 | 37.05 | 1.64 | 0.27 | 47.04 |
| *Cycas simplicipinna* | 1182 | 101 | 36 | 3 | 0.80 | 50 | 6.38 | 49.08 | 1.98 | 0.96 | 37.75 |
| *Cycas simplicipinna* | 1003 | 35 | 22 | 3 | 0.75 | 68 | 5.35 | 33.41 | 1.26 | 0.29 | 8.37 |
| *Cycas simplicipinna* | 930 | 15 | 21 | 3 | 0.80 | 60 | 5.73 | 47.30 | 2.31 | 0.37 | 13.54 |
| *Cycas simplicipinna* | 1251 | 170 | 35 | 3 | 0.75 | 80 | 5.72 | 41.40 | 1.68 | 0.30 | 23.85 |
| *Cycas simplicipinna* | 1340 | 52 | 20 | 3 | 0.70 | 60 | 5.75 | 38.53 | 1.57 | 0.30 | 16.24 |
| *Cycas simplicipinna* | 1056 | 210 | 23 | 3 | 0.60 | 60 | 5.44 | 46.58 | 1.93 | 0.38 | 11.52 |

**Figure S1** Distribution of ΔK from different K values from Structure analyses.


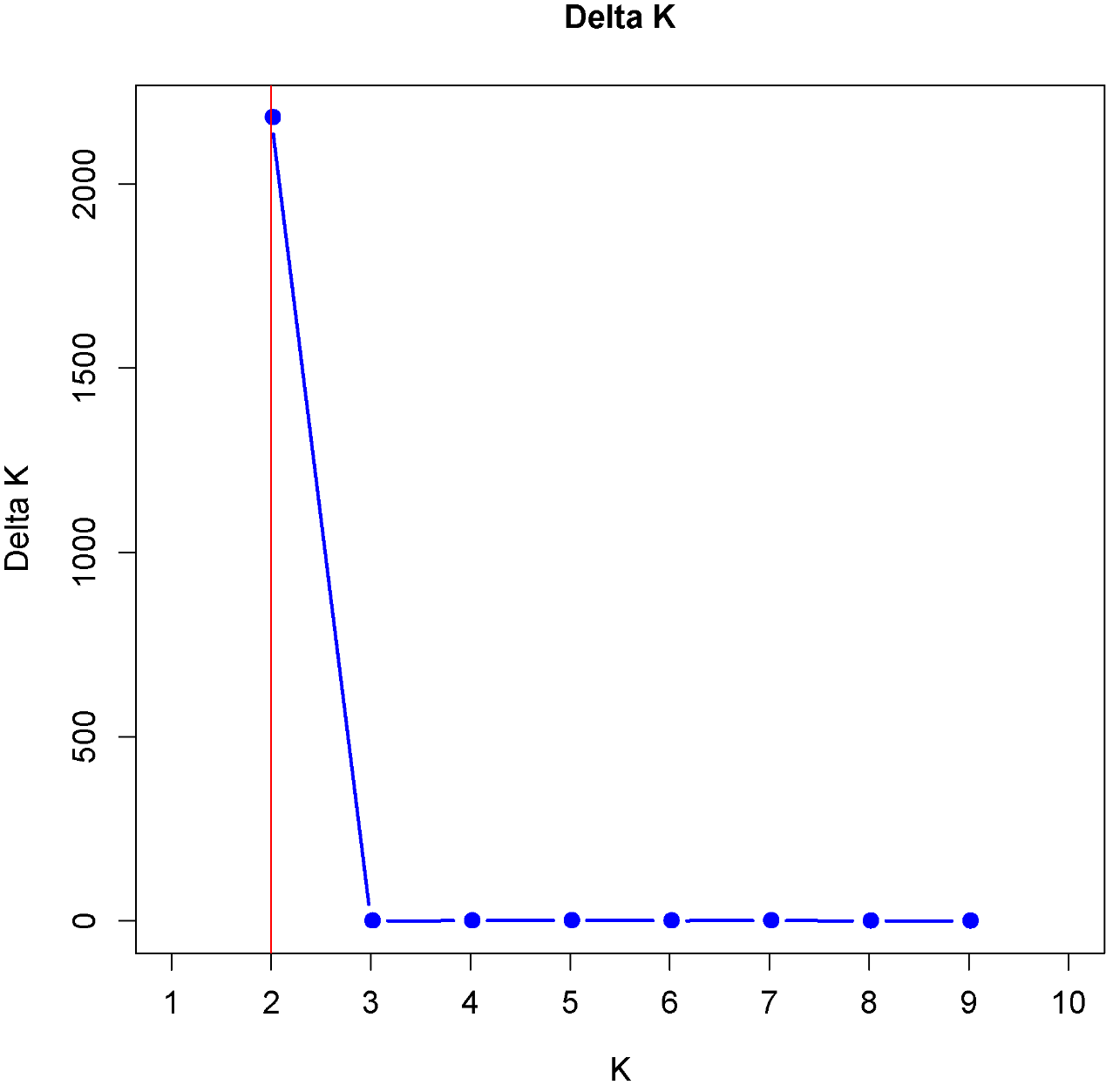


**Figure S2** Genetic structures of *C. simplicipinna* and *C. pectinata* based on 16 SSR loci as inferred by Structure analyses (K = 2).


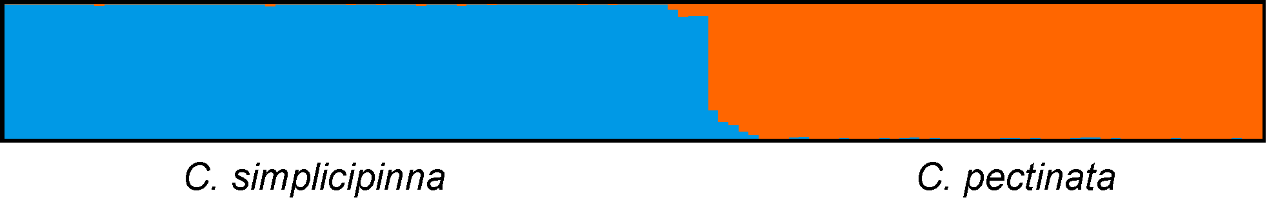


**References**

Cibrián-Jaramillo A, Marler TE, DeSalle R, Brenner ED (2008) Development of EST-microsatellites from the cycad Cycas rumphii, and their use in the recently endangered Cycas micronesica. Conservation Genetics, 9, 1051-1054.

Ju LP, Kuo CC, Chao YS, Cheng YP, Gong X, Chiang YC (2011) Microsatellite primers in the native perennial cycad Cycas taitungensis (Cycadaceae). American journal of botany, 98, e84-e86.

Li L, Wang Z-F, Jian S-G, Zhu P, Zhang M, Ye W-H, Ren H (2009) Isolation and characterization of microsatellite loci in endangered Cycas changjiangensis (Cycadaceae). Conservation genetics, 10, 793-795.

Wang YQ, Xiao SY, Xi, HH, Zhao ZC, Qiao YM, Song J, Wang ZC, Gong X (2021) Distribution status and habitat characteristics of three *Cycas* species in Southwest Yunnan. Journal of Plant Resources and Environment, 30, 36-43.

Wang ZF, Ye WH, Cao HL, Li ZC, Peng SL (2008) Identification and characterization of EST-SSRs and cpSSRs in endangered Cycas hainanensis. Conservation genetics, 9, 1079-1081.

Yang Y, Li Y, LI LF, GE XJ, Gong X (2008) Isolation and characterization of microsatellite markers for Cycas debaoensis YC Zhong et CJ Chen (Cycadaceae). Molecular ecology resources, 8, 913-915.

Zhang F, Su T, Yang Y, Zhai Y, Ji Y, Chen S (2010) Development of seven novel EST–SSR markers from Cycas panzhihuaensis (Cycadaceae). American journal of botany, 97, e159-e161.

Zhang M, Wang Z-F, Jian S-G, Ye W-H, Cao H-L, Zhu P, Li L (2009) Isolation and characterization of microsatellite markers for Cycas hainanensis CJ Chen (Cycadaceae). Conservation genetics, 10, 1175-1176.
